# Supplementary material for: Specific Glutamylation Patterns of the Cytoskeleton Confer Neuroresistance to Lobe X of the Cerebellum in a Model of Childhood-Onset Neurodegeneration with Cerebellar Atrophy
Source: Int J Mol Sci. 2025 Oct 25;26(21):10378. doi: 10.3390/ijms262110378 (PMC12610737; doi:10.3390/ijms262110378)
Supplement: Supplementary file 1 [file ijms-26-10378-s001.zip › Supplementary figure legends V2.pdf]

Supplementary figure 1. Heat map of gene expression. (A) Temporal expression of *Ccp1*, *Ccp4*, *Ccp6*, and *Ttll1* in vermis of wild-type animals (data corresponding to lobe X or lobes I-IX are represented separately). Fold change values of different ages are compared with those corresponding to P50 (grey). (B) *Ccp1*, *Ccp4*, *Ccp6*, and *Ttll1* expression in lobe X of wild-type mice compared with the rest of vermis from P20 to P50. (C) *Ccp4*, *Ccp6*, and *Ttll1* expression in lobe X of PCD mice compared with the rest of vermis at P20 and P25. (D) Relative expression of *Ccp4*, *Ccp6*, and *Ttll1* in PCD mice compared with wild type at P20 and P25. Colors represent the level of signification of fold change comparisons (set at $p<0.05$ at least): white, no significant; green, significant fold change > 1; light red, significant fold change < 1; dark red, significant fold change < 0.5.

Supplementary figure 2. Comparison of *Ccp1*, *Ccp4*, *Ccp6*, and *Ttll1* expression between lobe X and the rest of vermis in wild-type mice from P20 to P50. At P20 (A-D), lobe X showed lower expression levels of *Ccp1* (A) and *Ccp4* (B) genes compared to the other lobes. The other genes did not show differences (C, D). At P25 (E-H), lobe X showed lower expression levels of *Ccp1* (A) and *Ccp4* (B) genes compared to the other lobes. The other genes did not show differences (C, D). At P30 (I-L), the expression of *Ccp1* remained lower in lobe X compared to the rest of the lobes (A). However, *Ccp6* expression was significantly higher in lobe X (C). The other genes did not show differences (B, D). At P35 (M-P), *Ccp1* was expressed at lower levels in lobe X compared to the other lobes (A). *Ccp4* (B) and *Ccp6* (C) did not show significant differences, whereas *Ttll1* expression was significantly reduced in lobe X (D). At P40 (Q-T), significant differences were found only for *Ccp1* (A), which is expressed at lower levels in lobe X compared to the other lobes. The other genes did not show differences (B-D). Finally, at P50 (U-X), lobe X showed lower expression levels for all the genes in comparison with the rest of the vermis. Data are presented as the mean fold change ± standard error of the mean. * $p<0.05$; ** $p<0.01$.

Supplementary figure 3. CCP1, CCP6, TTLL1 and GADPH protein expression at P20 and P25 in wild-type and PCD animals: raw data. (A-C) Images corresponding to the molecular

weight marker. (A'-C') Raw Western blot images for all the proteins analyzed in this work at P20 and P25, in the different regions (lobe X or vermis, lobes –IX) and for both genotypes. Note that for figure B1 Western blot antibody stripping was performed and staining could be impaired, but in any case, the presence of proteins is confirmed.

Supplementary figure 4. CCP1, CCP6, TTLL1 and GADPH protein expression from P30 onwards in wild-type animals: raw data. (A-B) Images corresponding to the molecular weight marker. (A'-B') Raw Western blot images for all the proteins analyzed in this work from P30 to P50, in the different regions (lobe X or vermis, lobes –IX). Unspecif, unspecific.

Supplementary figure 5. CCP1 residual expression in PCD mice. These two images correspond to the same membrane but with different exposures. The left image (standard exposure, no overexposed bands) shows the presence of CCP1 in wild-type animals and its absence in PCD animals at P25. In the overexposed image on the right, faint bands corresponding to CCP1 can be seen in PCD animals, which may indicate residual expression.

Supplementary figure 6. Schematic representation of the functional network between the analyzed genes obtained with STRING database. *Ccp1* and *Ttll1*, which are directly involved in Cerebellar Purkinje cell differentiation, and *Ccp5*, *Ccp6* and *Ttll7* display closer functional associations (double lines).
