# Supplementary figures and images for: Specific Glutamylation Patterns of the Cytoskeleton Confer Neuroresistance to Lobe X of the Cerebellum in a Model of Childhood-Onset Neurodegeneration with Cerebellar Atrophy
Source: Int J Mol Sci. 2025 Oct 25;26(21):10378. doi: 10.3390/ijms262110378 (PMC12610737; doi:10.3390/ijms262110378)

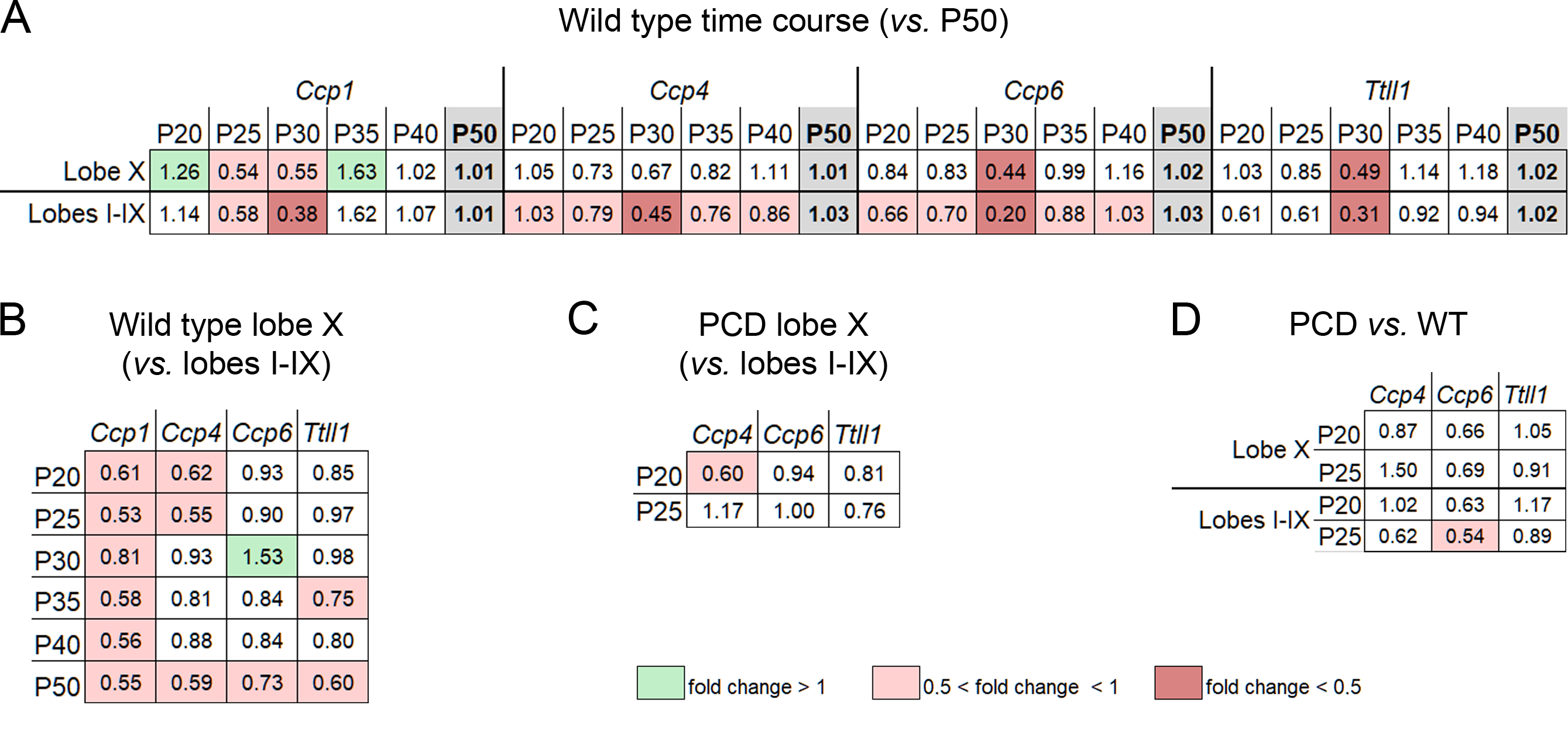

Supplement: Supplementary file 1 [file ijms-26-10378-s001.zip › Supp Fig 1.tif]

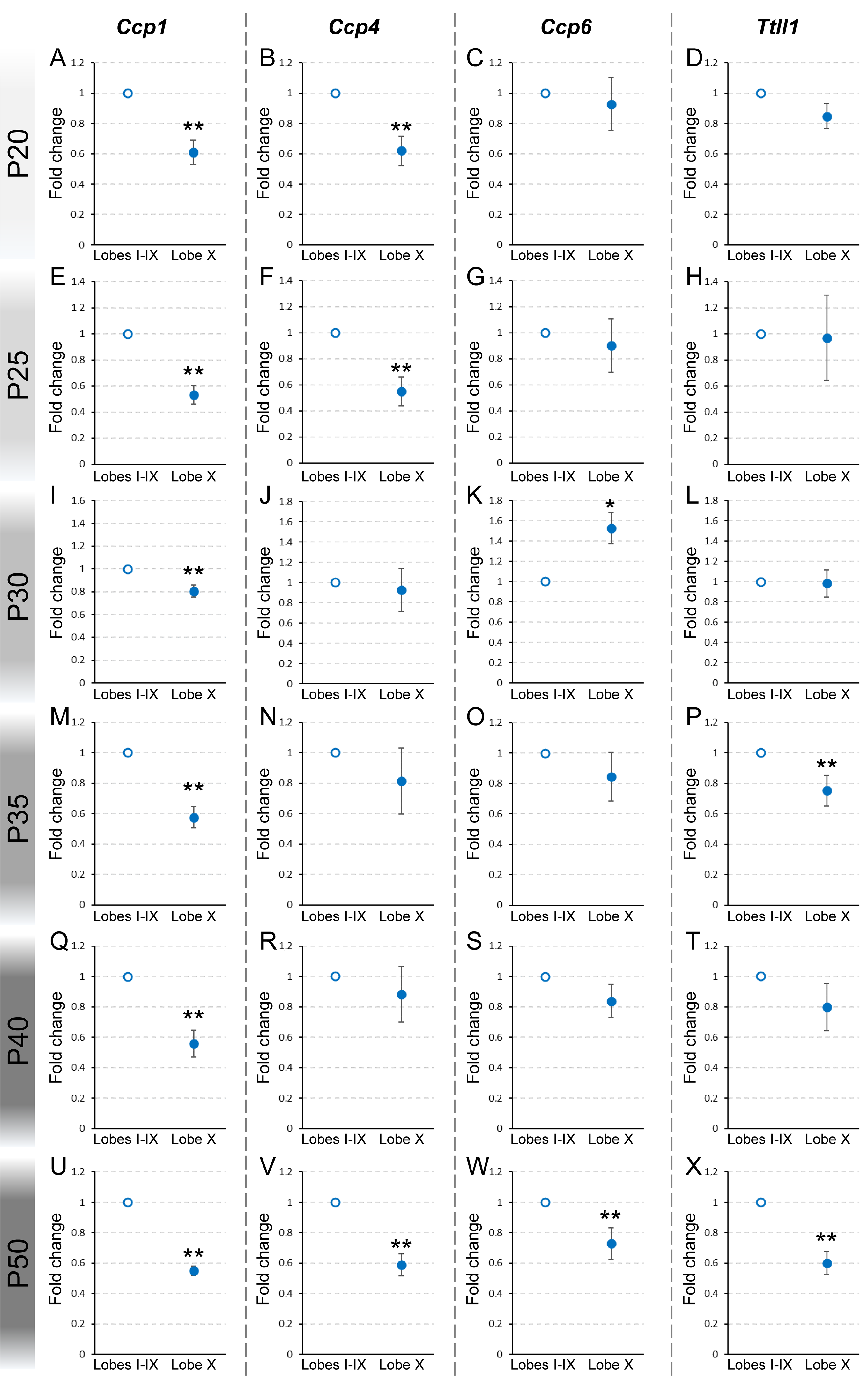

Supplement: Supplementary file 1 [file ijms-26-10378-s001.zip › Supp fig 2.tif]

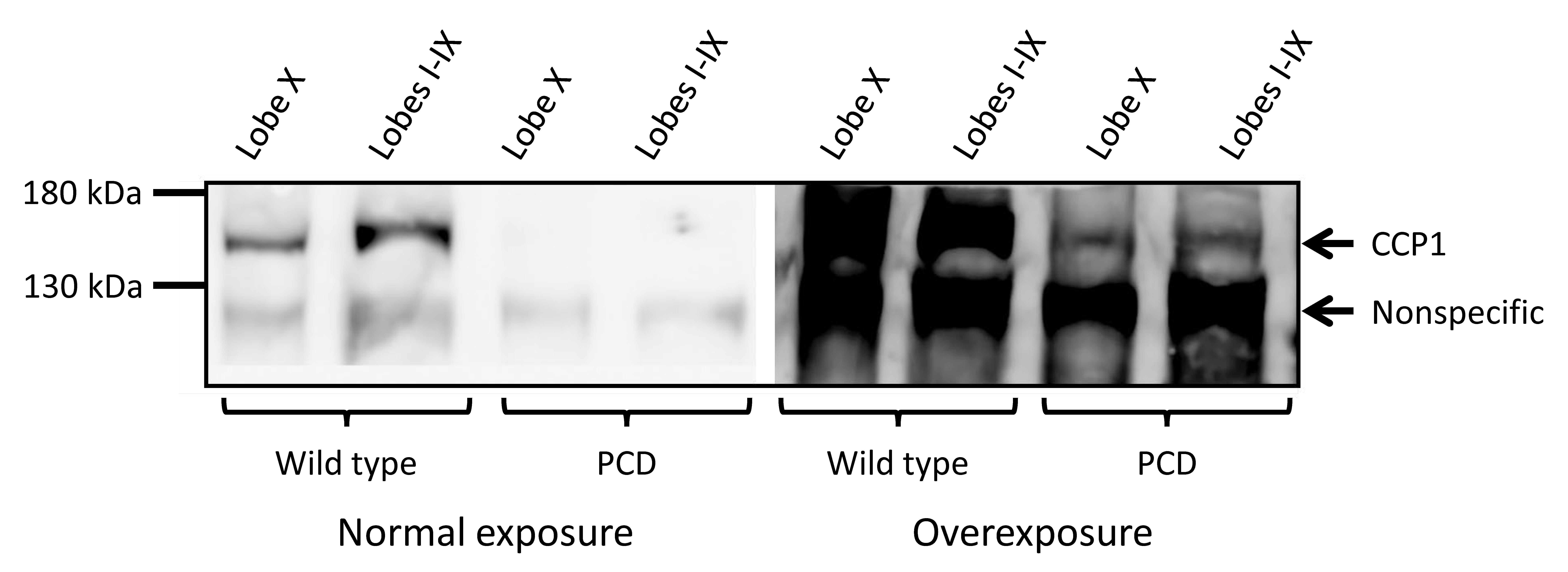

Supplement: Supplementary file 1 [file ijms-26-10378-s001.zip › Supp Fig 5.tiff]

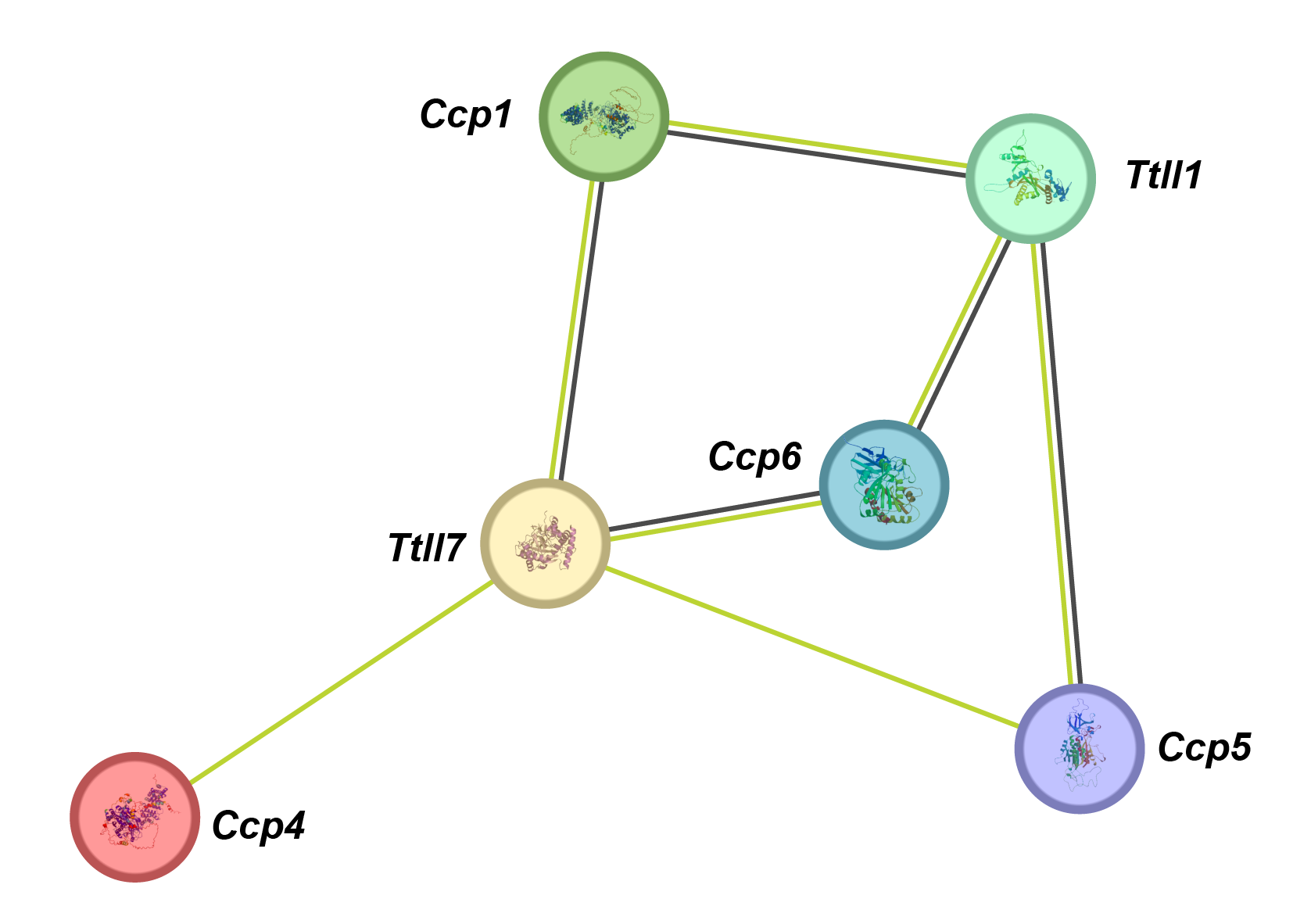

Supplement: Supplementary file 1 [file ijms-26-10378-s001.zip › Supp Fig 6.tif]
